# Nursing students' perception of patient‐centred care: A qualitative study

**DOI:** 10.1002/nop2.400

**Published:** 2019-10-17

**Authors:** Golnar Ghane, Maryam Esmaeili

**Affiliations:** ^1^ Medical Surgical Nursing School of Nursing and Midwifery Tehran University of Medical Sciences Tehran Iran; ^2^ Nursing and Midwifery Care Research Center School of Nursing and Midwifery Tehran University of Medical Sciences Tehran Iran

**Keywords:** content analysis, nursing student, patient‐centred care

## Abstract

**Aim:**

This study aimed to explain the understanding of nursing students from the concept of patient‐centred care.

**Design:**

This is an explorative and descriptive‐qualitative design.

**Methods:**

The participants consisted of 15 nursing students who were selected through purposeful sampling, and data were collected through in‐depth, semi‐structured interviews and analysed using a qualitative content analysis.

**Results:**

Data analysis led to the identification of three categories including the following: the inevitability of patient‐centred care, the patient‐centredness in comprehensive care and the importance of nursing process in patient‐centred care. Introducing students to the concept of patient‐centred care and how it can be achieved seems necessary during nursing education. Introducing a module on patient‐centred care to the nursing curriculum is suggested to familiarize students with this concept.

## 1 INTRODUCTION

Patient‐centred care has different aspects such as respecting patient's values and priorities, helping patients and providing emotional support, patient comfort, patient education, continuity and coherence of healthcare services, respecting patients, their family members and friends and participating them in the treatment process and ultimately having easy access to health care (Boer, Delnoij, & Rademakers, 2013; Cleary, 2016; Rathert, Wyrwich, & Boren, 2013). Thus, patient‐centred care is a valuable and comprehensive concept in nursing profession, which means to provide care with respect and to respond to preferences, needs and values of patient that include concepts such as patient‐centred communication, patient‐centred access, patient‐centred interview, patient‐centred resultant and patient‐centred diagnosis (Cleary, 2016; Weiner et al., 2013). Nurses in the healthcare system have a key role in proving patient‐centred care, which is a dynamic process between patient and nurse that occurs in various care settings (Ghane & Esmaeili, 2019; Cheraghi, Esmaeili, & Salsali, 2017; Ross, Tod, & Clarke, 2015). To achieve patient‐centred care, proper understanding and knowledge of care providers (nurses, students and experts) is essential and therefore the views of these people are very important in defining and understanding patient‐centred care (Bramley & Matiti 2014; Fayanju et al., 2016; Labrague et al., 2017).

Nursing educators should be aware of students' perceptions and views of educational concepts, such as care (Kermansaravi, Navidian, & Imani, 2013; Zamanzadeh, Valizadeh, Azimzadeh, Aminaie, & Yousefzadeh, 2014), to promote learning and reduce stress of students in clinical setting. Since the concept of patient‐based care is an essential component of nursing education, knowing the students' point of view, in addition to helping to facilitate their learning, empowers them in providing correct care to patient in the clinical setting (Loke, Lee, Lee, & Noor, 2015; Pai, Eng, & Ko, 2013). Students' understanding of patient‐centred care and the role of nursing education programmes are important in the teaching–learning process. Manninen, Scheja, Welin Henriksson, and Silén (2013) in regard to the students' self‐centred and patient‐centred experiences stated that self‐centred care for students is considered to be a tool for learning and a routine nursing practice. Therefore, it is vitally important to consider the concept of patient‐centred care in nursing education programmes (Manninen et al., 2013).

In Iran, limited studies have been conducted on the viewpoint and perception of nurses and patients about patient‐centred care (Cheraghi et al., 2017; Tafreshi, Pazargadi, & Abed Saeedi, 2007; Zamanzadeh, Azimzadeh, Rahmani, & Valizadeh, 2010). However, no study has ever been conducted on the understanding of nursing students from the concept of patient‐centred care. On the other hand, nursing students as the recipients of educational services and also the care providers are a good source of information for identifying existing problems, because they have direct and immediate interactions with the educational processes and clinical settings (Papathansiou et al., 2014). Therefore, this study aimed to explain the nursing students' perception of patient‐centred care.

## 2 METHODS

### 2.1 Aim and design

This is a descriptive‐qualitative study with content analysis approach. The purpose of this study was to determine the understanding of nursing students from the concept of patient‐centred care.

### 2.2 Participants

In this study, 15 nursing students (8 female and 7 male) participated in the individual interviews, and five students took part in two focus‐group interviews. Nursing students who were studying at Tehran University of Medical Sciences were selected through purposeful sampling. Students who were willing to participate in the study and had at least the experience of two terms of clinical education were enrolled in the study. To achieve the maximum variation, students from both sexes, different academic years and different grade point average were selected.

### 2.3 Data collection

Data collection was carried out using face‐to‐face, in‐depth and semi‐structured individual interviews. Data collection lasted for 6 months from October 2017–March 2018. The interviews were conducted in a quiet place at Tehran School of Nursing and Midwifery. Duration of the interviews varied between 25–45 min, depending on the students' willingness to continue with the interview. The interviews were conducted by the first author in Persian and then were translated into English. The interviews were recorded by a tape recorder and after each session, they were implemented word‐by‐word and analysed using MaxQDA‐10 software. The interviews continued until data saturate, which is a condition that new data or category is no longer obtained from the interviews. In the present study, in the eighth interview, the data were saturated and no new category was obtained. The following questions were the main interview questions: Can you explain about your care experience in clinical settings? Did you see any nurse who was delivering patient‐centred care during your clinical education? What measures taken by the nurses can lead to the provision of patient‐centred care? Have you heard about the concept of patient‐centred care? What do you think about it? Can you give us an example? In addition, exploratory questions were also used to clarify the responses of participants during the interview.

### 2.4 Ethical considerations

Proposals of this study were approved by the Ethics Committee of the Nursing and Midwifery School of Tehran University of Medical Sciences (IR.TUMS.FNM.REC.1397.013). The purpose and method of the study were explained to the students participating in the study, and they were told that they could withdraw from the study at any time, without having to face any fines or losses. An oral informed consent was also obtained from the students for participation in the study and recording of interviews. They were also assured about the confidentiality of their information and responses.

### 2.5 Data analysis

A content analysis approach was used to analyse the data. Content analysis as a research technique involves specialized techniques that are used to process scientific data. The qualitative content analysis reduces the data and gives them structure and order. The content analysis also contextualizes the symbolic meaning of messages (Vaismoradi, Turunen, & Bondas, 2013; Streubert & Carpenter, 2007). To analyse the data, recorded interviews were typed on paper word‐by‐word and read several times to obtain a general sense of understanding. Text of the interviews was also divided into meaning units that later were summarized and condensed. The abstract semantic units were labelled by codes and later, the codes were sorted into themes and sub‐themes according to their similarities and differences.

### 2.6 Trustworthiness

The accuracy of study is one of the important issues that should be considered at all stages of qualitative research, as it enables the reader to understand the events, effects and actions of the researcher (Elo et al., 2014). In this study, the peers' checking was used to ensure the validity of the findings. Data were independently coded and classified by the authors. Then, the themes resulted from the analysis were compared. In terms of disagreement on the themes, a discussion continued among the authors until a general agreement was reached. In this study, we also used member checking, so that a summary of extracted themes was given to several participants to be confirm by them. Accurate audit was also used in the early stages of the study and during data collection to ensure the reliability of the findings.

## 3 RESULTS

This qualitative study was conducted to determine the views of nursing students about patient‐centred care. The data were obtained by 15 individual interviews and two group interviews with participants that continued until data saturation. The mean age of the students participating in this study was 20.8 years and half of them were female and the other half were male. Also, they were studying at different educational terms, so that 5 students were at term 3, 4 students were at term 5, and 6 students were studying at terms 7 and 8.

The participants in this study expressed their views and experiences on patient‐centred care. According to the precise and in‐depth descriptions of the participants and analysis of data, three categories including the inevitability of patient‐centred care, the patient‐centredness of comprehensive care and the importance of nursing process in patient‐centred care were identified in this study. The second category consisted of two subcategories of patient education and communication and patient independence, which are described below along with the students' quotes:

### 3.1 The inevitability of patient‐centred care

Several nursing students participating in the study believed that care is essentially patient‐centred and cannot be done without considering the patient. They stated that the whole point of healthcare team is to address the needs and problems of patients and care should be patient‐centred, otherwise it would be meaningless. The students believed that patient should be the centre of all activities in the hospital and the presence of nurses in the hospital is only meaningful through the presence of patients. One of the nursing students studying at term 7 stated:

There is no such a thing as the patient‐free care, so all care should be patient‐centred.

From the viewpoint of this student, care is essentially patient‐centred and without patient it is meaningless. Students believed that all efforts of nurse should be in line with the needs and priorities of patients and no shortcoming in this area is acceptable. They believed that the main policy in hospital development should be around the patient‐centredness, but some problems and obstacles may lead to a diversion from this policy, where case the problems are related to the approach of healthcare and treatment teams, including nurses. Several students referred to the clinical accreditation and clinical governance in hospitals and believed that, patient‐centredness is the core of clinical governance. However, based on their experiences in clinical settings, the students believed that emphasis is placed more on recording and documentation rather than the patient and nurses are more involved with paperwork and this causes them to pay less attention to patients in hospitals. A nursing student in regard to the emphasis being placed on recording in the audit stated:

You must record everything you do and if you don't, you are in trouble in audit. Due to the lack of time, you may record it without actually given any education to patient, so that in the accreditation and audit they think you have done it. It means that, you have record this just to keep your job or because of accreditation and in reality, you have not had any time to do it, so this is the documentation‐centered care, not the patient‐centered care.

From the viewpoint of students, patient‐centredness is an integral part of nursing care:

Naturally, we can say we have no patient‐centred care where there is actually no care. (Nursing student of term 7)

From the viewpoint of this participant, care is patient‐centred and it has no meaning without the centredness of patient. In spite of this perception, some students did not understand the concept of patient‐centred care. They believed that familiarity with these concepts should be realized during the course of study and the ways of achieving patient‐centred care were not clear for them during their education. One of the students at term 6, in this regard, stated:

I became familiar with the family‐centered care when I went to a pediatric internship, where I realized how important the family is. But now, I hear this new concept of patient‐centered care and I'm not very familiar with it.

### 3.2 Patient‐centredness in the provision of comprehensive care

Nursing students defined the patient‐centred care as caring for patient taking into account all aspects of the patient. They believed that nurses should pay attention to all physical, psychosocial, spiritual, social and cultural aspects of patient while they deliver care and in their view, a care is comprehensive when it considers all aspects of the patient. According to the understanding of nursing students, comprehensive care includes patient communication and education and preserving patient's independence in decision‐making. They believed that all of these measures should be provided for patient together and absence of one undermines the concept of patient‐centred care:

Patient‐centered care is to consider all physical, psychological and mental needs of patient and provide the best comprehensive care for him/her. (Student nursing at term 5)

#### 3.2.1 Patient education and communication

When the students were asked to recall an experience of patient‐centred care during their clinical education, their main focus was on patient communication and education. They remembered the practice of nurses who had established a good relationship with the patient and educated them by effective communication. The students have considered those nurses as a model for patient education. From the nursing students' perspective, patient communication is one of the main principles and examples of patient‐catered care:

All care should be patient‐centered. Education is also important and we should all have it. If this is not being done, it is our fault. The education, care and communication must be patient‐centered. (Nursing student at term 3)

According to the understanding of nursing students, provision of care along with proper communication and education about unknowns to patients increases patient satisfaction and is an example of patient‐centred care. Nursing students referred to nurses’ control over the situation and keeping patient calm as the art of nursing and believed that a nurse who could establish a proper communication with all individuals, including patient and treatment team, is a successful nurse in providing patient‐centred care. A nursing student in regard to her clinical education experience stated that:

There was a nurse in one of the wards who was communicating really well with the patients, physicians and students, I mean that, she explained the situation to patients and talked about drugs' side effects and complications due to her extensive experiences… She managed to calm patients when they were angry. (Nursing student at term 3)

#### 3.2.2 Patient independence in decision‐making

The participating nursing students referred to paying attention to the viewpoints of patients as an example of patient‐centred care. They believed that in the care process, patient's trust should be gained during patient communication and education. Patient‐centred care is a care that considers the patients' bill of rights and patient's independence in decision‐making as one of the items of the bill. As students, they believed that any measure taken for the patients should be explained to them and their consent should be obtained and no patient should be treated without his/her consent. One student in this regard said:

The main patient's rights should be considered and patient's opinion should be asked. Also, despite the explanations, if patient does not want to receive a particular care, his/her view should be respected even if it is contrary to the opinion of doctor and nurse. (Nursing student at term 3)

Students in their experiences referred to cases where nurses and doctors did not pay any attention to the needs and satisfaction of patient. They considered a care that does not have any regard for patient's rights, a none patient‐centred care, and believed that sometime care is given to patient regardless of patient‐centredness due to many barriers. One student recalled her experiences and stated:

When the patient is ready for operation, they first coordinate and document his file without asking his consent or explaining anything to him about the operation…… They also give the file to patient's family and ask them to go and stamp it. Well, all these indicate that patient is not the priority. (Nursing student at term 5)

### 3.3 The importance of nursing process in patient‐centred care

Some of the students stated that patient‐centred care depends on the implementation of nursing process. They believed that accurate and timely assessment of patient helps to identify and prioritize patient's needs and make plans and implement them according to the identified needs. Students considered the initial assessment of the patient as the first step in the implementation of patient‐centred care:

Patient‐centered care is a care that ranges from assessment to follow‐up (five stage of nursing process) and is focused on patients and their need and condition assessments. (Nursing student at term 5)

Nursing students considered patient‐centred care as a care that includes final assessment of performance. They stated that, at the end of nursing process, care interventions should be monitored and evaluated. From their point of view, evaluation in the care process determines the satisfaction of patient and achievement of the goals set to meet the patient's needs:

Care should be based on the needs and condition of patient and then it should be evaluated correctly. A clear framework should also be determined to see whether we have reached our goals or not. (Nursing student at term 3)

## 4 DISCUSSION

From the perspective of nursing students in this study, the underlying core of patient‐centred care is the comprehensiveness of care that must be provided inevitably through nursing process. This comprehensive care includes three important components of education, proper communication with patient and observance of patient's independence. Jouzi, Vanak, and Mohammadi (2013) from the viewpoint of students and teachers in this study stated that patient‐centred care is the humanistic care that covers many aspects such as communication with patient and his family, having a humanistic view of the patient, providing scientific care based on the patient's needs and conditions and maintaining patient safety( Jouzi et al., 2013). Other studies have also suggested that patient‐centred care is considered a moral and fundamental value for nurses, which is related to the satisfaction of patient and care provider, better health processes, higher quality of care and provision of a more effective care (Cheraghi et al., 2017; Fayanju et al., 2016; Rathert et al., 2013; Weiner et al., 2013). McCormack, Treiman, et al. (2011) provided a general framework for patient‐centred care from the perspective of patients and staff in six levels, including appropriate clinical relationship with patient, giving information to patient, recognizing and responding to patient's feelings, managing uncertainty, decision‐making and activating patient self‐care (McCormack, Treiman, et al., 2011). Although limited studies have been conducted on the views of students about patient‐centred care, the results of all of them are consistent with each other and show a positive attitude towards patient‐centred care, so that students believe patient‐centred care can facilitate nurse's performance in the clinical setting (Jouzi et al., 2013; Rathert et al., 2013; Weiner et al., 2013). From Watson's point of view, care has four essential components, including respect for person's identity and value, recognizing the individual's unique response to the disease, supporting the individual's independence and helping the person to achieve his/her maximum ability (Watson, 1999). This is in line with the findings of present study.

Correct patient education is one of the most important principles of patient‐centred care from the viewpoint of students. They considered the nurses' training role as one of the most important roles of nurses in providing comprehensive patient care. Studies have also reported that proper health education to patient improves patient's independence and self‐care, reduces complications, increases patient satisfaction and reduces the costs (Weiner et al., 2013; Zamanzadeh et al., 2010). In a study by Gotlarzi et al, 84% of students emphasized that patient education is one of the main duties of nurses (Gotlarzi, Ahmadvand, & Farajollahi, 2004). The results of this study are consistent with the findings of Taheri et al study, where the students considered patient education to be the primary duties of nurses (Tahery et al., 2011). However, some studies have shown that patients are inadequately trained in the clinical setting and emphasized that nurses and students should take responsibility for patient education and provide patients with appropriate training and education (Baraz, Memarian, & Vanaki, 2015; Boer et al., 2013).

In this study, students have also emphasized that effective communication is essential in patient‐centred care as it creates a sense of security and trust in patients, facilitates interpersonal relationships between treatment teams and increases patient and family satisfaction. From the perspective of students, nurses must have appropriate communication skills in addition to other clinical skills. Communication skills that are most important to nurses include providing information, asking questions, establishing a friendly relationship with patient and building partnerships and obtaining patient's cooperation. An effective communication skill in nurses results in obtaining sufficient information from patients, correct diagnosis, gaining patient trust and, ultimately, successful treatment. In this regard, Jouzi et al. (2013) argued that appropriate communication in patient‐centred nursing interventions would create a sense of respect and optimism in patients and allows them to have a more respectful relationship with nurses. It can also create a sense of hope about the effectiveness of treatment (Jouzi et al., 2013). From the perspective of nurses, Ross, Tod, & Clarke (2014) reported that nurses' appropriate communication with patient, family and treatment team is essential in providing patient‐centred care, establishing friendly relationships and building trust in patient, which are the main aspects of patient‐centred care (Ross et al., 2014).

Correct relationship with patient involves allocating time to hear the patient and his family's concerns, providing patient with the necessary information and helping to reduce patient's anxiety and stress. The participants also reported that patients and their families would be more satisfied with patient‐centred care services and proper responses of nurses. In a study by Jouzi et al. (2013), the participants emphasized on the need to pay attention to patients, families and their concerns and tried to focus on patients' specific conditions while communicating with them and meeting their needs (Jouzi et al., 2013). In addition, students in the present study and the study of Ross et al (2014) believed that effective communication between members of treatment team, a relaxed but at the same time professional approach in reducing the stress of all individuals and satisfaction of patient and his family are essential for patient‐centred care (Ross et al., 2014). Therefore, involving patients and their families during care decision‐makings and establishing appropriate communication during the implementation of nursing interventions should be considered.

Patient‐centred care is an approach that establishes appropriate communication with the patient, involves patients with their treatment and well‐being by providing them with sufficient health information and has a direct relationship with the promotion of health care, experiences and positive outcomes (McCormack, Treiman, et al., 2011). However, despite the importance of effective communication, the participants in this study and most other studies believed that relationship between treatment team and patient is more task‐focused and patient‐centred relationship is much less common. Most nurses do not communicate well with patients, and their communication approach focuses more on routines and procedures than patient care and assessment (Ghane & Esmaeili, 2019; Rathert et al., 2013).

The importance of patient's independence and satisfaction from the services was another aspect of patient‐centred care from the viewpoint of students in this study. They believed that, despite the importance of patient's independence in the process of hospital accreditation and audit, it is often ignored during the care delivery. Other studies from the patient's viewpoint have reported that patient autonomy is often ignored in the care process, so that most patients believe that nurses respect their autonomy far less in two aspects; the amount of information given to them and their participation in the decision‐making process (McCormack, Treiman, et al., 2011; Zamanzadeh et al., 2010). Students and nurses also believed that patient's independence during nursing care is not well‐respected (Tafreshi et al., 2007). Ross et al (2014) refer to the importance of patient's desire and involvement in decision‐making as the principles of patient‐centred care. Therefore, educating nursing staff about the importance of patient's independence is essential in nursing care.

Students in our study believed that patient‐centred care is inevitable, and care should be patient‐centred. They also believed that this is not happening currently in the hospitals. They stated that they have not seen patient‐centred care in their clinical education experience. Nursing students and nurses in the study of Ross et al (2014) believed that patient‐centred care is a prerequisite for the patient care (Ross et al., 2014). In this regard, studies have also shown that patient‐centred care should be provided for all patient groups. They have also referred to the application of patient‐centred care in clinical settings with the focus of managers being placed on the importance of patient‐centred care (Weiner et al., 2013; Ghane & Esmaeili, 2019). Thus, patient‐centred care is a comprehensive and multiple care and its realization in everyday nursing in hospital environment is a challenge. Thus, it requires more attention from managers to create appropriate ground for the provision of patient‐centred care.

At the end, nursing students in this study considered patient‐centred care as a care that includes final evaluation of performance, which should be implemented based on the steps and principles of nursing process. Nursing process is a problem‐centred, dynamic and patient‐oriented process and teaching the applications of nursing process to nurses can help to improve the quality of nursing and patient‐centred care (Tafreshi et al., 2007). Students in Jouzi et al (2013) study emphasized on the provision of scientific care based on the patient's needs and conditions and the importance of applying scientific methods, such as the use of nursing process in patient care (Jouzi et al., 2014).

## 5 CONCLUSION

Results of this study revealed the features of patient‐centred care from the perspective of nursing students. However, despite the important role of nursing students in clinical settings and patient care, most students referred to the lack of patient‐centred care education in their educational curriculum. Therefore, patient‐centred care education as a separate unit/module should be added to the student's educational curriculum. It should also be added to the continuing education programmes of nurses who can help to establish patient‐centred care in the healthcare system. This study only attempted to explain the nursing students' perception of patient‐centred care. Further studies are suggested to examine the role and place of nursing education in preparing students to improve the quality of care. Furthermore, a quantitative study with the title of "assessing the impact of nursing process training on achieving patient‐centered care" can also help to explain the importance of nursing process in improving the quality of care.

### 5.1 Limitations

Students with the experience of clinical education were used in this study. Perhaps, the use of first or second term students with no experience of clinical education in hospital environment could provide more data. Another limitation was the use of students who were not doing student work to help us extract their pure experiences.

## 6 IMPLICATION FOR NURSING PRACTICE

Introducing a module on patient‐centred care in the nursing curriculum is suggested to familiarize students with this concept.

## CONFLICT OF INTEREST

No conflict of interest has been declared by the authors.

## ETHICAL APPROVAL

This research project has been approved by a research ethics committee of the Tehran University of Medical Science (TUMS) (Number: IR.TUMS.FNM.REC.1397.013).
